# On the characteristics of landslide tsunamis

**DOI:** 10.1098/rsta.2014.0376

**Published:** 2015-10-28

**Authors:** F. Løvholt, G. Pedersen, C. B. Harbitz, S. Glimsdal, J. Kim

**Affiliations:** 1 Norwegian Geotechnical Institute, PO Box 3930 Ullevål Stadion, 0806 Oslo, Norway; 2 Department of Mathematics, University of Oslo, PO Box 1053 Blindern, 0316 Oslo, Norway

**Keywords:** tsunamis, landslides, numerical modelling

## Abstract

This review presents modelling techniques and processes that govern landslide tsunami generation, with emphasis on tsunamis induced by fully submerged landslides. The analysis focuses on a set of representative examples in simplified geometries demonstrating the main kinematic landslide parameters influencing initial tsunami amplitudes and wavelengths. Scaling relations from laboratory experiments for subaerial landslide tsunamis are also briefly reviewed. It is found that the landslide acceleration determines the initial tsunami elevation for translational landslides, while the landslide velocity is more important for impulsive events such as rapid slumps and subaerial landslides. Retrogressive effects stretch the tsunami, and in certain cases produce enlarged amplitudes due to positive interference. In an example involving a deformable landslide, it is found that the landslide deformation has only a weak influence on tsunamigenesis. However, more research is needed to determine how landslide flow processes that involve strong deformation and long run-out determine tsunami generation.

## 1. Introduction

Earthquakes have caused more than 80% of all documented historical events [1] and a cautious estimate indicates that at least 24 million people may be exposed to their induced tsunamis of low-frequency recurrence [2]. Landslides, including volcano flank collapses or volcanically induced flows, constitute the second-most important cause of tsunamis [3]. Owing to their visual presence, subaerial rock slides have long been recognized as tsunami sources, and studies at least go back to Wiegel 1955 [4]. Submarine landslide tsunamis, however, were not fully recognized prior to the 1998 Papua New Guinea (PNG) event [5]. This was despite clear tsunami evidence from events such as the 1929 Grand Banks tsunami [6,7], the 1979 Nice tsunami [8], as well as the 8150 BP Storegga landslide tsunami [9,10]. In fact, the PNG event gave rise to a scientific dispute, eventually leading to a general acceptance that the tsunami was due to a slump source [11,12]. Past investigations may consequently not fully recognize submarine landslides as possible triggers, and particularly older historical records are therefore likely to be biased [3].

Tsunamis induced by landslides display a greater variety depending on their origin compared with earthquakes. Sources range from events of local character [13–15], to large-volume landslides with long run-out and substantial regional impact [16,17]. Generation mechanisms are also more diverse, spanning from impulsive waves due to subaerial landslides hitting the water with high impact velocities (e.g. [18–20]) to submerged landslides moving farther but at lower speeds (e.g. [21]). The dating of older events represents another challenge [22], and complicates the use of landslide statistics for estimating the landslide-induced tsunami hazard (e.g. [23,22]). Owing to the above reasons, the nature and hazard posed by landslide tsunamis are not as well understood as those from earthquakes.

Recent analysis relates landslide triggers to their geographical position and geophysical setting, as well as to features of the geological age. Urgeles & Camerlenghi [24] presented a database of landslides as well as a landslide zonation for the Mediterranean, suggesting that landslides emerging from tectonically passive margins may involve larger but more infrequent maximum volumes, whereas the landslides in tectonically active margins are smaller in size and more frequent. Solheim *et al.* [25] and Lee [26] suggested that recurrence of landslides offshore the Norwegian and eastern US coastlines are heavily linked to glacial–interglacial cyclicity. Furthermore, Masson *et al.* [27] suggest that the massive landsliding from the Canary Islands are linked to early island formation. Urlaub *et al.* [28] investigated statistics of sea-level high- and low-stands, but found no significant correlation with generation of large landslides. Whereas the studies cited above provide explanations and statistics for past landslides, we are still far from being able to use them to derive firm probabilities for their induced tsunamis.

The strongly varying landslide initiation, gradual mass mobilization [29] and dynamics make the quantification of the tsunamigenesis from submarine landslides demanding. Although there exists considerable evidence of many pre-historic events across the world documenting the run-out (e.g. [24,27,30–32]), evidence revealing the kinematics of submarine landslides remains scarce. However, the 1929 Grand Banks [6,7], 1945 Makran [33] and 1930 Orkdalsfjorden [34] are notable exceptions as they involved cable breaks and hence some possible evidence for the landslide evolution. Further examples of possible landslide tsunamis involving cable breaks are discussed in [35]. Another well-studied example is the 1998 PNG event caused by a submarine slump, where the run-up heights have been well reproduced through numerical modelling (e.g. [11–13,36]). However, well-documented cases are limited in numbers and do not cover the variety of processes that are of expected importance for landslide tsunamigenesis.

The above introduction sets landslide tsunamis into the context as a complex hazard that depends on the interplay between different triggers, materials, scales and mechanisms. The nature of the submarine landslides have previously been reviewed by many (e.g. [37–42]) and is therefore not the primary subject here. Moreover, the geographical extent of previous landslide induced tsunamis and the hazard they pose are also treated elsewhere (e.g. [3,22,43]). Here, we attempt to shed light on how the interplay between basic landslide kinematics and wave propagation govern tsunamigenesis. The primary emphasis is on fully submerged landslides, treated through a literature review mixed with new results, while tsunamis due to subaerial landslides are discussed in a more rudimentary way, primarily citing existing literature. This paper provides a significantly updated review compared to similar past reviews (e.g. [44,45]), with a stronger focus on modelling and mathematical aspects of the tsunami generation.

This paper is organized as follows: §2 discusses the various models for simulating landslides and tsunamis, as well as their coupling. In §3, an in-depth analysis of tsunami generation from fully submerged blocks are given, to demonstrate the basic properties of tsunami generation. We separate the analysis into translational landslides with long run-out and more impulsive events such as slumps, and we also study the relative effects of frequency dispersion. Section 4, exemplifies the effects of landslide deformation, first for a retrogressive landslide, and second for a tsunami induced by a deformable granular landslide. In §5, we review recent findings related to subaerial landslide tsunamis, focusing on laboratory measurements.

## 2. Modelling approaches

The dynamics of submarine landslides is complex, involving transformation of the intact matter through remoulding, fluidization, deposition and erosion. Often, the flowing masses may involve strong mixing and possible development of both a dense flow part and a dilute (turbidity) current. The dense flow provides the majority of the rapid water volume displacement, which eventually governs tsunamigenesis (the interface shear contributes less, see e.g. [9,21]). The subsequent discussion is therefore limited to tsunamis induced by dense flows. The most general procedure for modelling the tsunami generation is to use the hydrodynamic equations in primitive form (without simplifying assumptions) in three dimensions (e.g. [46–48]). Simplifying assumptions of these equations include the layered depth averaged Navier–Stokes formulation [49–51].

As an example of a primitive coupled tsunami-landslide model, we follow [52] and write the Navier–Stokes equations for the conservation of volume and momentum a phase *n* in a two-phase landslide water mixture:
2.1
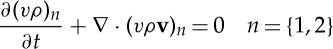
and
2.2
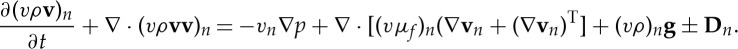
In the equations above, we denote the relative volume fraction *v*, the density *ρ*, velocity vector **v**, pressure *p*, dynamic viscosity *μ*_*f*_, gravity **g** and an inter-phase momentum coupling term **D** (the sign depend on *n*). Gauer *et al.* [52] used this two-phase model with a strain-softening landslide rheology to study the last phase of the Storegga Slide in two-dimensional geometry. The problem was however too demanding to resolve the full three-dimensional extent of the landslide due to the complex flow field for the dense, clay-rich landslide rheology. The same is likely to be the case for granular models, and depth-averaged models are therefore most often employed to model long run-out landslides.

Here, we present the Voellmy model [53] as an example of a depth-averaged landslide model. The Voellmy model is formulated in terms of a bed-parallel coordinate (here denoted *x*′). The Voellmy model assumes hydrostatic pressure (and a uniform velocity profile) and Coulomb friction over a slowly varying topography. It is described by the equations
2.3
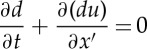
and
2.4
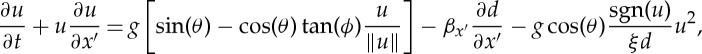
where *d* denotes the total landslide flow depth (the term landslide thickness is also used below), *u* the depth-averaged velocity, *β*_*x*_ an internal pressure term taking into account whether or not a fluid element is subject to passive or active stress, *ϕ* the friction angle and *ξ* a turbulent dissipation coefficient. Neglecting the *u*^2^ term, we obtain the celebrated Savage–Hutter model [54]. The *u*^2^ term is however useful as it allows for improved representation of the granular flow as well as adding hydrodynamic drag.

Another popular depth-averaged landslide model is the Bing model [55], which uses a Bingham rheology. The Bing model differs from the granular Voellmy and Savage–Hutter models in that the flow is divided into a bottom shear layer and a top plug layer, among others. Whereas the Bing model is more realistic for clay-rich landslides than the simplest granular landslide models [56], it involves some limitations that have been addressed recently. To this end, more general formulations including additional terms such as hydrodynamic drag, transportation of blocks, remoulding and hydroplaning have been presented (e.g. [57–59]). Here, the two former terms limit the landslide speed, and the latter two are needed to explain the extreme run-out distance on gentle slopes of large landslides such as Storegga.

While the shallow water wave equations are often sufficient for simulating ocean-wide earthquake tsunami propagation, frequency dispersion is more important for landslide tsunamis [60]. To this end, Boussinesq models are often applied (for a review, e.g. [61–63]). A dimensionless (scaled) set of Boussinesq equations may be written
2.5
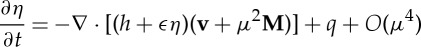
and
2.6
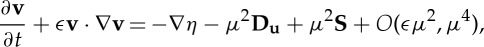
where *η* is the surface elevation, *h* the water depth and **v** is the current velocity. The higher order flux term **M** and momentum term **D**_**u**_ depend on the form of the Boussinesq formulation. The dimensionless parameters *ϵ* and *μ* introduce scales for amplitudes and wavelengths, respectively. The Boussinesq models have been implemented using a variety of numerical schemes, most recently using approximate Riemann solvers in combinations with TVD limiters [64–67]. In the Boussinesq model employed for demonstrating tsunamigenesis below, we may switch between standard dispersion properties (such as e.g. [68]) or higher order dispersion (e.g. [69]). By neglecting all dispersive terms of *O*(*μ*^2^), equations (2.5) and (2.6) correspond to the nonlinear shallow water equations. In equations (2.5) and (2.6), the primary source terms are introduced by the *q* term in the continuity equations, whereas the second-order source terms in the momentum equations are denoted by **S**. When the characteristic lengths of the source are much larger than the depth *q* may be expressed as *q*=−∂*h*/∂*t*. However, when length scales of the order of the depth, or shorter, are present, the surface response is effectively distributed over a few depths. If the ocean depth is constant and *d*/*h*≪1 we may write
2.7
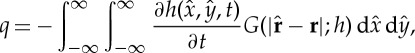
where the representation of *G* is fulfilling the linear full potential equation, thus acting to prevent the smallest wave components to be conveyed from the seabed to sea surface. Expressions for *G* are given in [70,60]. Since *G* vanishes rapidly with increasing argument we may use this formula also for a gently sloping seabed. On the other hand, neither such source representations, nor depth-integrated models as such, can describe violent impacts of subaerial landslides with flow separation and complex subsequent flow patterns. Apart from such cases the source representations as outlined above are convenient as they enable a general input from different landslide models, such as [53,55] through one-way coupling. The two-way coupling may be neglected in many situations with submerged landslides (e.g. [71]).

## 3. Tsunamigenesis due to submerged blocks

The generation and propagation of tsunamis due to landslides have been subject to substantial analyses, through laboratory scale measurements, analytical models and numerical modelling. They differ from earthquake tsunamis in their extent and typical wave characteristics (see [45,3] for a discussion). Owing to the different scales involved from local subaerial landslides with large impact velocities to huge submarine landslides, methods and indicators to characterize the waves may differ substantially. Commonly, however, the extent of the landslide (defined by a typical length ℓ, width *w* and thickness *d*) and the dynamics (defined by the typical velocity *u* and acceleration *a*), as well as the linear shallow water (LSW) wave celerity 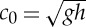 are used to characterize tsunamigenesis. Table 1 lists some relevant scaling relations from the literature, including the Froude number *Fr* that measures criticality in the tsunami generation, the relative landslide thickness *S*, a related Froude number *Fr*_ls_ for the landslide, a time scale *τ* for the degree of frequency dispersion, and finally amplitude characteristics for the maximum scaled crest elevation *η*_c_ introduced by Hammack [72]. Below, the tsunami characteristics for different types of landslides under idealized conditions are briefly discussed.

**Table 1.** Some scaling relations relevant for landslide tsunamigenesis. *t*_c_ is the critical time scale for the duration of the seabed displacement introduced by Hammack [72], *T* represents a typical wave period, *h* the undisturbed water depth, *d* the landslide thickness and *g* the acceleration of gravity. Additional quantities are explained in the main text.

explanation	formula	reference/use
tsunami Froude number	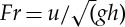	[18,73]
landslide Froude number	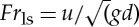	
scaled landslide thickness	*S*=*d*/*h*=(*Fr*/*Fr*_ls_)^2^	[18,73]
dispersion time scale	*τ*/*t*=6*h*/*gT*^3^	[60,70,74]
Hammack's crest elevation	*η*_c_/*h*∝*d*ℓ/[*h*⋅*c*_0_⋅*t*_c_]	[72]

Here, we consider a rigid rectangular block with volume *V* =*d*ℓ*w*, density *ρ*_*s*_, total block mass *m*_*s*_=*ρ*_*s*_*V* and added mass *m*_*w*_=*ρ*_*w*_*V*_*w*_. We define the added mass coefficient by *C*_*m*_=*m*_*w*_/*m*_*s*_. The block starts from rest on an inclined plane with slope angle *θ*, with a Coulomb friction coefficient *f*=tan(*ϕ*) and surface skin friction *C*_F_, and moves with a variable velocity *u* downslope. Following [9,75], we may write the equation of motion
3.1
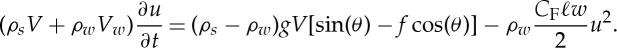
Solving equation (3.1) we get the following expressions for *u* and *a* in terms of the terminal velocity *u*_*t*_, 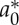 the initial acceleration and the characteristic time 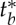 [75,76]:
3.2
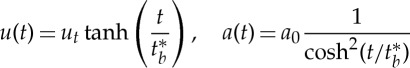
and
3.3
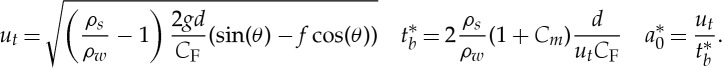
In a series of examples given below, we demonstrate tsunamigenesis employing a sine-shaped velocity profile for the motion of a block. The velocity profile demonstrates tsunami generation due to basic kinematic landslide parameters such as the velocity and acceleration
3.4
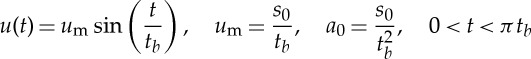
Here, *u*_m_ is the maximum block velocity, *a*_0_ the initial acceleration, *s*_0_=*R*/2 a typical travel distance (*R* being the run-out distance), and *t*_*b*_ a typical time scale for the motion (*πt*_*b*_ is the total landslide running time).

**Figure 1.**
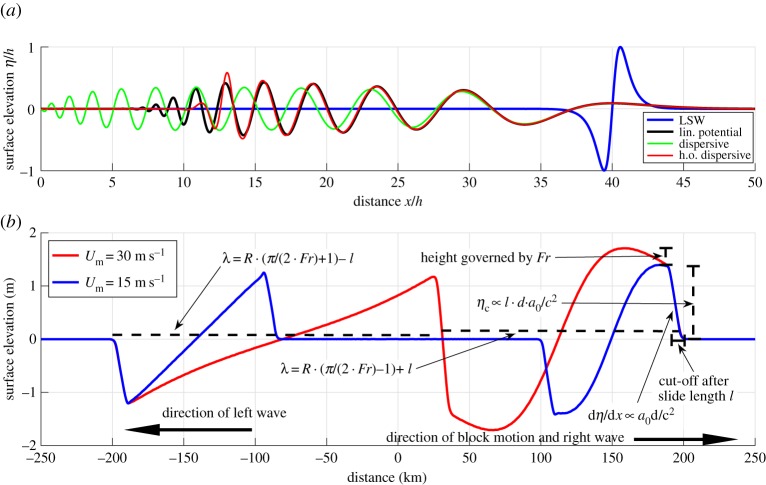
(*a*) Normalized plot of the evolution from a dipole-shaped initial surface deformation. The time is 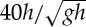 and the linear shallow water (LSW) solution reflects the shape of the initial condition. Note that the output is scaled. For all the long-wave models, the full potential source filter is applied for modelling the tsunami generation. (*b*) Simulated surface elevation using an LSW model and a moving rectangular block with length ℓ=8000 m and thickness *d*_0_=100 m as the source, using two different velocity profiles; *t*_*b*_=256 *s*, *u*_m_=15.3 m s^−1^ and *Fr*=0.13 in blue; *t*_*b*_=512 s, *u*_m_=30.6 m s^−1^ and *Fr*=0.25 in red. The initial acceleration is in both cases *a*_0_=0.06 m s^−2^. We use this simulation to explain basic aspects of the tsunami generation under the LSW assumption, as indicated by arrow boxes and simple drawings.

Since many of our examples are conducted on a constant depth *h*, we choose typical values for *u*_m_, *a*_0_ and *t*_*b*_ to demonstrate how the landslide kinematics influences the tsunami generation, rather than obtaining them directly from equations like (3.3). Based on the analysis of [77] using equation (3.4) for the block velocity, assuming low Froude numbers and large run-out to landslide length ratio, we obtain a simple expression for the leading crest or trough wave elevations *η*_c_ for a plane wave on constant depth induced by a landslide under linear hydrostatic conditions:
3.5
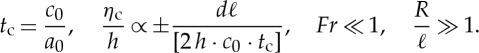
In the above equations, we recognize the critical time scale derived by Hammack [72] in table 1. The sign depends of the direction of the wave (positive for the wave in the direction of the landslide, negative for the wave in the opposite direction). According to Watts [76], *t*_c_ is related to the characteristic time for the wave generation. Combined, equation (3.5) and table 1 show that a small *t*_c_ enables build-up of larger initial crest heights, and that *t*_c_ becomes small for the larger initial accelerations. Further, *η*_c_ scales linearly with block dimensions ℓ and *d*. Haugen *et al.* [77] explained how the *η*_c_/*h* expression in equation (3.5) is due to the leading elevation originating from the front of the landslide being cut off from the depression originating from the tail. Efficient build-up of the wave is enhanced through a high initial acceleration, enabling a higher wave before cut-off. For higher Froude numbers, or more impulsive landslide motion (smaller *R*/ℓ), the expression in equation (3.5) is invalid. Some basic aspects relating to the findings of [77] are shown in figure 1 and elaborated further below.

We note that when the run-out to landslide ratio *R*/ℓ<4*Fr*/(*π*∓2*Fr*), the leading waves due to the front and rear volume displacements will no longer interact. In this case, we have an impulsive condition, and the surface elevation in each direction of propagation is given by
3.6
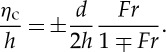
For derivations and further discussion, including the analysis of the forced wave following the landslide, see [78,79]. While this expression was originally derived assuming a constant slide speed with instantaneous start and stop, it will also hold for a variable landslide velocity. We also note that while equation (3.5) derived for translational (long run-out) landslides relates *η*_c_/*h* primarily to the acceleration and slide length, we obtain a dependency of the Froude number in equation (3.6) for the more impulsive condition (short run-out). As shown below however, the above expressions for *η*_c_/*h* become too simplified when frequency dispersion is prominent. The same is the case when prominent nonlinearities are present, as for subaerial landslides (see §5).

Short horizontal scales and sharp gradients in the landslide geometry and impulsive motion of the landslide represent challenges for traditional depth-averaged tsunami models, assuming that wavelengths extend over multiples of the mean water depth. In the following, we investigate tsunami generation from a short and impulsive landslide on constant depth using two different models.

First, we use a linearized version of the GloBouss model [20,80,81] under the LSW, standard dispersion and higher order dispersion assumptions, respectively. To represent the landslide source in GloBouss, we use the full potential representation enabled through equation (2.7) that effectively filters the shortest horizontal depth scales in the tsunami generation. Second, a full potential model based on the Boundary Integral formulation [82] is employed in linearized form. The latter model is used as a reference model to check the accuracy of the long-wave model (GloBouss). For plane waves on constant depth equation (2.7) gives a Green's function 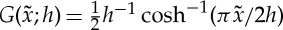. A block slide corresponds to a moving point source and sink, at the front and rear, respectively. The extreme case of a short block slide (ℓ/*h* small) then yields a source distribution *q*=*c*_0_*Fr*ℓ*d*(d*G*(*x*−*x*_*f*_(*t*);*h*)/d*x*), where *x*_*f*_ is the landslide position. A similar source distribution will arise from, for instance, a slump with short run-out.

We observe that the source strength is proportional to ℓ, implying that the length of a short block slide mainly affects the amplitude of the generated waves. Furthermore, if we assume that the duration of the event is short, and the run-out small, the wave generation will correspond to imposing an initial surface elevation *η*=*C*(d*G*/d*x*), where *C*=ℓ⋅*d*⋅*R*. We note that in the limit of a fully impulsive landslide, we obtain a different source strength than the one found by Haugen *et al*. [77] for a landslide with longer duration. The resulting wave is also the shortest any bottom disturbance may generate, and its evolution may thus yield a conservative assessment of the applicability of wave models.

In figure 1, the simulated surface elevation using both GloBouss and the reference model is depicted. We observe that the leading crest is small and that the higher order Boussinesq model combined with the source filter follows full potential theory well, apart from at the end of the wave train where errors due to the non-zero minimum group velocity inherent in the higher order (h.o.) model become apparent. The standard Boussinesq model, on the other hand, exaggerates the dispersive wave-train. It must be noted that this is the worst case concerning the performance of long-wave models. Longer slides will produce a smaller fraction of short waves and their tsunamis are better reproduced by the Boussinesq models. The favourable comparison with the reference model allows us to use the GloBouss model in our further analysis of tsunami generation and propagation, in all cases in combination with the full potential Green function representation for landslide sources [70].

Next, we demonstrate plane wave tsunamigenesis for simple non-deformable landslides on constant depth using equation (3.4) for the landslide block motion. The landslides start from rest at *x*=0 and move rightwards. In most of the examples, we use a rectangular block with thickness *d*_0_ as the landslide source; however a secant hyperbolic shape mimicking a slump (similar to the one proposed by Grilli & Watts [83]) is also used for comparison:
3.7
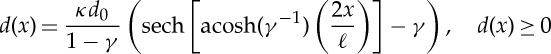
Here, we use constants *γ*=0.717 and *κ*≈1.56, the former taken from [83], the latter chosen to give identical slide volume with the rectangular block. Compared to the rectangular block, the slump shape combined with *R*/ℓ≪1 essentially mimics a rotational failure.

**Figure 2.**
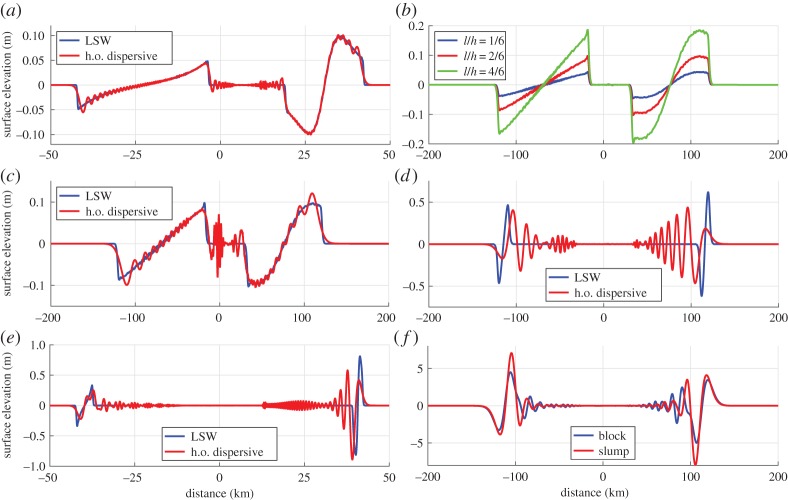
Plane evolution of waves generated by rightwards moving blocks on a constant depth sea-floor. In all cases, the landslide motion has terminated, and the relative landslide thickness is *S*=*d*/*h*=0.067. (*a*) Rectangular block source, landslide and depth parameters ℓ/*h*=1.67, *t*_*b*_=256 s and *u*_m_=15.3 m s^−1^, *Fr*=0.40 (*h*=150 m) and *a*_0_= 0.06 m s^−2^, LSW simulation (blue) and higher order dispersive simulation (red). (*b*) LSW model using a rectangular block source, landslide and depth parameters *t*_*b*_=256 s, *u*_m_=15.3 m s^−1^, *Fr*=0.13 (*h*=1500 m), *a*_0_=0.06 m s^−2^, for 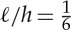, 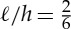 and 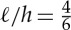, respectively. (*c*) Rectangular block source with the landslide and depth parameters 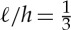, *t*_*b*_=256 s and *u*_m_=15.3 *m* s^−1^, *Fr*=0.13 (*h*=1500 m) and *a*_0_=0.06 m s^−2^, LSW simulation (blue) and higher order dispersive simulation (red). (*d*) Rectangular block source with the landslide and depth parameters, 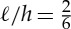, *t*_*b*_=32 s and *u*_m_=15.3 *m* s^−1^, *Fr*=0.13 (*h*=1500 m) and *a*_0_= 0.48 m s^−2^, LSW simulation (blue) and higher order dispersive simulation (red). (*e*) Rectangular block source, landslide and depth parameters ℓ/*h*=1.67, *t*_*b*_=32 s and *u*_m_=15.3 m s^−1^, *Fr*=0.40 (*h*=150 m) and *a*_0_=0.48 m s^−2^, LSW simulation (blue) and dispersive simulation (red). (*f*) Higher order dispersive simulation with the landslide and depth parameters ℓ/*h*=10.67, *t*_*b*_=32 s and *u*_m_=15.3 *m* s^−1^, *Fr*=0.13 (*h*=1500 m) and *a*_0_= 0.48 m s^−2^, rectangular block (blue) and slump (red).

Figure 1*b* demonstrates basic principles of tsunami generation under the LSW assumption for two different velocity profiles. They have the same initial acceleration *a*_0_=0.06 *m* s^−2^, but have different maximum Froude numbers *u*_m_/*c*_0_ and run-out distances. We focus on the wave moving rightwards in the same direction as the landslide, and first recognize that the steepness of the wave front is the same for the tsunami originating from both velocity profiles, proportional to *a*_0_*d*. At a distance ℓ behind the wavefront, the steepness changes (see also figure 2*b*). The change in steepness is caused by the depression wave from the rear part of the landslide that starts to interact with the wave originating from the front, which may reduce the surface elevation. This restricts further increases of *η*_c_ for *x*<190 km in the case of *Fr*=0.13, while the elevation for *Fr*=0.25 is not immediately cut off and a slightly larger *η*_*c*_ is obtained due to the more critical generation (higher Froude number).

Figure 1*b* further shows that the larger landslide velocity (and run-out) mainly has the effect of increasing the wavelength. Owing to critical wave generation effects, we observe also a Doppler shift, i.e. the wavelength *λ* is shortened for the wave moving in the direction of the landslide and stretched in the opposite direction.

Figure 2*a* shows a similar example but with a more critical wave generation (*Fr*=0.40). Compared to the previous example, we first observe a stronger Doppler shift, seen as a larger difference in wavelength for the opposite directions of propagation. Secondly, we also see that the increased Froude number leads to a larger difference in *η*_c_ in the two directions of propagation. We note that in the present example, dispersion is found to be of minor importance. Figure 2*b* shows results from LSW simulations with a relatively long duration of motion (*t*_*b*_=256 s), and where the landslides have short block lengths (ℓ<*h*). In this case, *η*_c_ increases linearly with the block length, which is due to the cut-off proportional to ℓ and the steepness proportional to *a*_0_ as explained in our first example (figure 1). As *c*_0_*πt*_*b*_≫ℓ, both wavelengths remains essentially unaffected by ℓ, and rather depends on the landslide duration, e.g. *λ*≈*c*_0_⋅*π*⋅*t*_*b*_. The surface elevation in figure 2*c* is simulated using one of the blocks from the latter example, showing that the importance of dispersion is relatively small.

Next, we employ an impulsive velocity profile giving a block that produce shorter and higher waves, with more apparent frequency dispersion (shown in figure 2*d*). The degree of dispersion at the termination of the motion may be analysed through the dispersion time in table 1, noting that the landslide duration and typical wave period are similar. This gives dispersion times *τ*=0.0014 (negligible dispersion) for the long landslide duration and *τ*=0.09 (pronounced dispersion) for the impulsive landslide, respectively (see [60] for discussion of the *τ* parameter). For the case of the long landslide duration, dispersion is still visible despite the low *τ*. This is interpreted as a result of the short frequency components in the steep wavefronts.

In figure 2*e*, waves originating from an impulsive landslide with a higher Froude number are depicted. Owing to a pronounced Doppler effect, a clear difference in wavelength for the waves moving in the opposite directions is evident. As the landslide motion is impulsive, the waves become short, but due to the Doppler effect the degree of dispersion is much more pronounced for the shorter wave moving rightwards (in the direction of the landslide).

We compare the waves generated by a slump and a rectangular block in figure 2*f*. For the present example, we see that the slump source is a more efficient generator than the rectangular block (this difference will not appear in the limit of a short landslide). In the last example, we use *R*=980 m and ℓ=16 km, implying *R*/ℓ<4*Fr*/(*π*+2*Fr*). Under this condition, the maximum crest elevation is determined by the Froude number rather than the acceleration. We also note that Tappin *et al.* [12] propose a length ℓ≈4.5 km and velocity *u*_*m*_≈15 m s^−1^ for the 1998 PNG event, which would imply *R*/ℓ=0.2, 4*Fr*/(*π*+2*Fr*)=0.15 and 4*Fr*/(*π*−2*Fr*)=0.18, meaning that interaction between the waves due to the front and rear part of the landslide are present, but small. For the 1998 PNG case, *η*_c_/*h* will depend on a combination of *Fr* and *a*_0_ as *R*/ℓ≈4*Fr*/(*π*±2*Fr*). In the cases of short run-out, dispersion is often prominent, which adds to the complexity.

## 4. Retrogression and stretching

**Figure 3.**
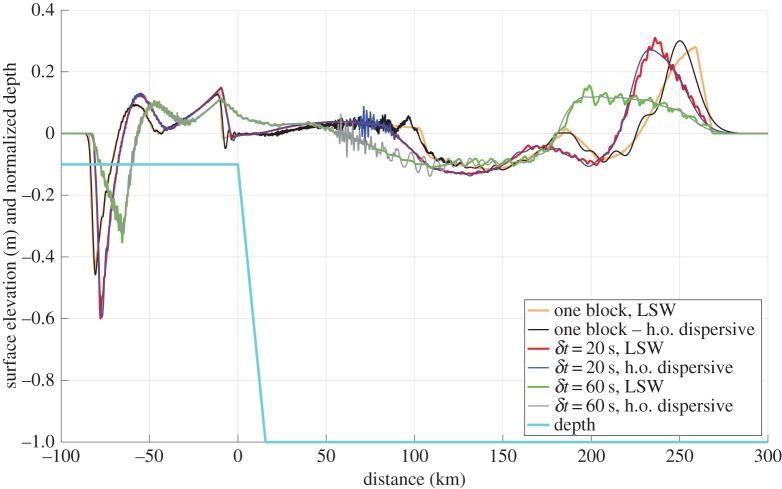
Plane wave tsunami generation by a retrogressive landslide moving downslope. Landslide kinematics is given by equation (3.4), with *t*_*b*_= 512 s and maximum horizontal speed *u*_m_=30.6 m s^−1^. Three cases are considered, a single block (zero time lag), a time lag of *δt*= 20 s, and a time lag of *δt*=60 s. The depth is scaled by the maximum water depth of 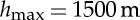.

Below, we demonstrate the effect of landslide retrogression and landslide deformation on a simple shelf geometry in one horizontal dimension. The bathymetry consists of two constant depth parts of *h*=150 *m* (left) and *h*=1500 m (right), respectively, divided by a 4.92° constant slope (figure 3). In both examples, we use a rectangular block with length of ℓ=4000 m as a reference source. We also add a cosine function with a half wavelength of 500 m at both ends of the block. Smoothing of both the block and the retrogressive landslide (see below) was applied to reduce the shorter components in the wave spectrum that are subject to critical wave generation. The landslide block thickness is set to *d*_0_=10 m.

First, we simulate the tsunami generation from the rectangular block moving with pre-scribed motion according to equation (3.4) with *t*_*b*_=512 s and maximum horizontal speed *u*_*m*_=30.6 m s^−1^, meaning that the landslide obtains its maximum velocity when it hits the deep plain. The leftmost part of the block is located at *x*=0, and the block moves rightwards. Using this simulation as a reference, we subdivide the above block into nine equal parts having the shape
4.1
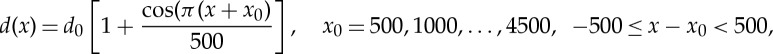
where *x*_0_ represents the incremental shift of the centre of each block, while *d*_0_=5 m. The frontal block is released first, while the release time for the subsequent blocks are delayed with a constant time lag *δt*. The simulated surface elevations under the LSW and higher order dispersive assumptions are depicted in figure 3 at *t*=2200 s, after the landslide has come to rest. In the present case, dispersion mainly smooths the irregular wave that appear due to the multi-staged release. In fact, the leading wave from the single block appears as more dispersive than the wave originating from the retrogressive landslide. Figure 3 hence demonstrates that retrogression may have the effect of stretching the tsunami compared to the single block, and in the case of large time lags, reducing its amplitude. For shorter time lags, however, positive interference from the waves originating from individual blocks increases the maximum amplitude (particularly the leading trough) for the leftward wave. This has also been reported previously by Haugen *et al*. [77] using an analytical model, but in the present case the use of a numerical model allows simulating a more general example with variable depth. We note that the limited effect of dispersion in this example is due to the applied velocity profile. Using a more sophisticated retrogressive landslide model leading to larger accelerations, Løvholt *et al*. [84] found a strong effect of dispersion.

**Figure 4.**
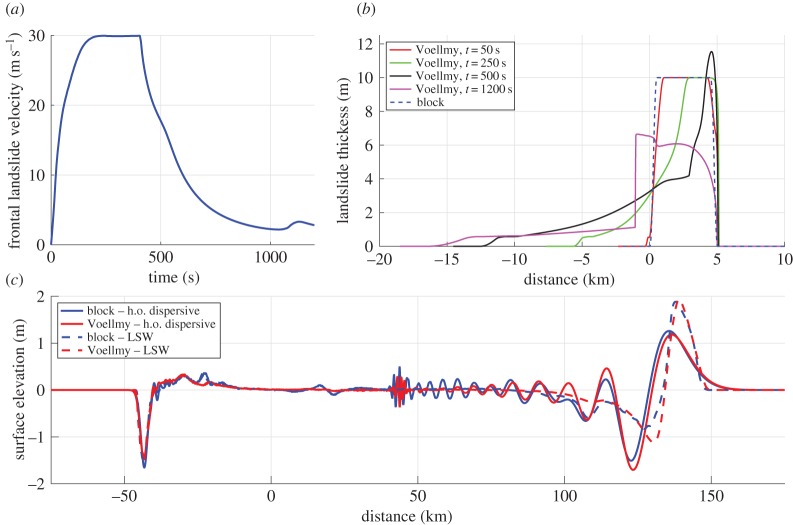
(*a*) Velocity of the slide front using the Voellmy model. (*b*) Shape of the deforming landslide for different times. In the figure, we have shifted the frontal slide position to correspond with the one from the block, the purpose is to compare how the deforming landslide deviates from the block with time. (*c*) Simulated tsunami after *t*=1200 s. We note that the short frequency waves at *x*≈40 km are unphysical, but that they do not affect the main wave system.

In the second example shown in figure 4, we use the Voellmy model [53] to calculate the time-dependent depth *h*(*t*) as the tsunami source term. We tuned the Voellmy landslide parameters *ϕ*=1.5° and *ξ*=1500 m s^−2^ to provide a maximum landslide velocity of about *u*_m_=30 m s^−1^. The resulting velocity of the front of the landslide as well as the deformation pattern is shown in figure 4*a*,*b*. In figure 4, we have fixed the frontal slide position to *x*=5 km to demonstrate how the landslide deforms as a function of time compared to the initial conditions. We also use the reference block with the same kinematics as the deforming landslide as shown in figure 4 as an alternative source term. This means that the front of the block and the front of the deformable landslide move with the same speed.

The simulated surface elevations at the end of the simulation is shown in figure 4. We see that the rightward moving wave is significantly affected by dispersion, while the leftward moving wave generated in shallower water is adequately described by the LSW model. More remarkable, however, is the similarity between the tsunamis due to the rigid block and the deformable landslide simulated by the Voellmy model. In the present case, most of the wave generation takes place during the early phase of the motion with high acceleration at shallow depth (involving also higher Froude numbers), and during this phase the deformation of the landslide is limited. In particular, the front remains unaffected during early stages. Hence, we conclude that rapid deformation during the initial acceleration phase is generally needed to influence the tsunamigenesis.

We do not claim that the result in figure 4*c* is general, but note that for the most effective tsunami generators, those with large acceleration, there is limited time for the landslide to deform during the acceleration phase, and in this example the use of a block source is used fairly successfully. However, acceleration may be reduced by added mass effects (due to ambient water being accelerated with the landslide), and this effect is not included in the present example. In a previous study by Watts [85], the added mass reduced the acceleration up to about 30%.

## 5. Tsunamigenesis due to subaerial landslides

Subaerial landslides may impact water bodies at high speed. In contrast to waves generated by fully submerged landslides, subaerial landslides often involve large Froude number and nonlinearities. Their generation has been studied in a range of experimental investigations. Some selected recent studies are listed in table 2. As a representative example to demonstrate tsunamigenesis from subaerial landslides, we review basic findings from the two-dimensional experiments of [93,18]. In their experiments, flow separation formed due to the violent impact when 
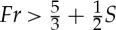
 [93]. Fritz *et al.* [18] further defined a linear relation (typically *a*_1_−*b*_1_*S*>*Fr*>*a*_2_−*b*_2_*S*, where *a*_1,2_ and *b*_1,2_ are constants) to group the downstream wave system. The groups range from a nonlinear oscillatory wave train (smaller *Fr* and *S*) through a nonlinear transitional wave system, to eventually a solitary like wave and a breaking bore (higher *Fr* and *S*). This description of the wave system was partly verified later by a solid block model [73], with the difference that the block provided solitary waves for lower values of *Fr* and *S* than the granular landslide by Fritz *et al*. [18]. We observe that in all cases, the wave system inherently involves strong components of either frequency dispersion or nonlinearity, most commonly both. Furthermore, Fritz *et al*. [18] obtained a good correlation of the maximum crest height *η*_c_ for
5.1
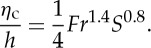
We now compare the above equation with *η*_c_/*h* for submarine landslides in equation (3.6), assuming a small Froude number ([1−*Fr*]≈1). We obtain 
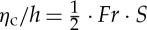
 from (3.6). We remark that this equation (5.1) only holds when the front and rear waves are not interacting. Then, we see a weaker influence of *S* on *η*_c_ in the subaerial case, but a stronger influence from the landslide impact velocity expressed by *Fr*. When the Froude number increases, *η*_c_/*h* will exhibit a nonlinear dependency of the Froude number also for the submarine landslide-induced wave. We add that for many of the three-dimensional cases listed in table 2, a smaller degree of nonlinearity is obtained for *η*_c_/*h* due to the amplitude reduction from radial spread. Three-dimensional generation also enables near-field edge waves, for discussions see e.g. [19,88]. Gentler waves are also encountered in the special case of the experiments by Lindström *et al.* [92] conducted in a 1 : 500 fjord geometry representing a branch of the fjord Storfjorden in Western Norway.

**Table 2.** A list of some selected experiments investigating wave parameters due to subaerial landslides. The studies [18,86–89] all conducted statistics for the maximum crest elevation *η*_c_/*h*, with an impressive correlation coefficient range of *R*^2^≈0.91±0.03.

study	landslide	geometry
Huber & Hager [90]	block	3D-radial evolution
Fritz *et al.*[18]	granular	2D
Panizzo *et al.*[86]	block	3D-radial evolution
Di Risio *et al.*[87]	block	3D-conical island
Sælevik *et al.*[73]	block	2D
Fritz *et al.*[91]	granular	2D-Litya Bay geometry
Mohammed & Fritz [88]	granular	3D-radial evolution
Heller & Spinneken [89]	block and granular	2D and 3D
Lindström *et al.*[92]	block	3D-fjord geometry

Owing to their violent impact, primitive models are often needed to simulate tsunami generation due to subaerial landslides; early applications include among others [94,95]. Gisler *et al.* [47] simulated the three-dimensional generation of the tsunami due to a hypothetical landslide emerging off La Palma Island using the hydrocode SAGE. Løvholt *et al.* [20] extended the simulations to the ocean-wide scale by coupling the SAGE simulations to the dispersive GloBouss model (see above). Owing to the strong interplay between a long dispersive wave-train and bathymetric refraction, Løvholt *et al.* [20] illustrated an extremely complex propagation pattern. These Boussinesq simulations clearly revealed that extrapolation of the early asymptotics of the near-field wave attenuation either from numerical simulations or from laboratory experiments could gravely underestimate the expected wave amplitudes in the far-field. Løvholt *et al.* [20] have further shown that the leading wave tsunami amplitudes due to subaerial landslides decay more slowly (by *r*^5/6^) than those from fully submerged landslides (by *r*^7/6^). While the studies of [47,20] involved an extreme and unlikely landslide volume of 375 km^3^ for the hypothetical case of a La Palma tsunami, Abadie *et al.* [48] used the VOF model Thetis to simulate the tsunami due a series of smaller landslide volumes emerging from La Palma. Giachetti *et al.* [96] compared the simulated tsunami run-up due to a pre-historical event on Tenerife with heights obtained from paleotsunami investigations, concluding that a staged release was necessary to fit the observations. Multi-staged release is supported also by offshore observations [97,98]. The far-field propagation of tsunamis induced by rock-slides in fjords has somewhat different characteristics compared to offshore events, such as the case reviewed above, as multiple reflections make the propagation more complex. A recent simulation, using the scaled laboratory set-up by Lindstrøm *et al*. [92], showed that the wave propagation along the fjord clearly involved frequency dispersion, but only to a moderate extent [99].

## 6. Conclusion

Landslide tsunamis exhibit a large variety according to their source origin. While tsunami amplitude due to subaerial landslides is often characterized by the frontal area of the landslide and its impact velocity, tsunami generation due to submerged landslides depends on the time evolution of the landslide. The initial acceleration is the most important kinematic landslide parameter, determining the initial elevation for the case of long run-out. However, when the run-out distance is sufficiently short compared with the slide-length, such as for subaerial landslides and slumps, the landslide Froude number becomes the most important kinematic parameter for determining the maximum initial elevation. In such cases, the landslide motion is impulsive, generating shorter and higher waves, leading to frequency dispersion. The flow of landslides over large distances may be quite complex. Retrogression, or multi-staged release of the landslide mass, an effect that is common for both subaerial and submarine landslides, complicates the process further. Retrogression has the main effect of stretching the induced wave compared to a the generation due to an intact block. Currently, new model development within this field is taking place [84], and we expect to obtain new knowledge about retrogressive generation mechanisms in the coming years. For tsunami generation due to a landslide flow model, we observed that the deformation process was too slow to influence the tsunami generation significantly. More research is however needed to explore the generality of this result; it is probable that landslide deformation may prove important for the tsunami amplitude in other examples.

We presently see a clear knowledge gap related to linking landslide volume to its tsunamigenic potential, mainly through providing more accurate estimates for landslide parameters such as acceleration and velocity. More research is needed to include reliable landslide models into tsunami analysis. For submerged landslides, conventional Boussinesq-type models can be used to simulate tsunami generation and propagation even when we encounter short horizontal scales introduced by the landslide. To capture the latter effect, full potential filtering of short-wave components emerging from landslide water volume displacement is needed. For subaerial landslides, the main challenge is related to modelling the violent impact and initial wave generation, and more general (primitive) models are needed. While a rich literature exists on laboratory investigations due to impact tsunamis, the numerical models remain, however, less mature. However, recent applications [47,48] shows that this branch of research is progressing as well.
